# Characterization of the soft-tissue wall lining residual periodontal pockets and implications in periodontal wound healing

**DOI:** 10.1007/s00784-023-05122-y

**Published:** 2023-07-24

**Authors:** Evangelia Gousopoulou, Athina Bakopoulou, Dimitrios Laskaris, Epameinondas Gousopoulos, Danae A. Apatzidou

**Affiliations:** 1 Department of Preventive Dentistry, Periodontology & Implant Biology, School of Dentistry, Faculty of Health Sciences, Aristotle University of Thessaloniki (A.U.T.H.), 54124 Thessaloniki, Greece; 2 Department of Prosthodontics, School of Dentistry, Faculty of Health Sciences, Aristotle University of Thessaloniki (A.U.T.H.), 54124 Thessaloniki, Greece; 3 Department of Molecular Pathology, Netherlands Cancer Institute, 1066CX Amsterdam, Netherlands; 4 Department of Plastic Surgery and Hand Surgery, University Hospital Zurich, 8091 Zurich, Switzerland

**Keywords:** Gene expression profiling, Gingival biopsies, Periodontal granulation tissue, Mesenchymal stem cells, Wound healing, Periodontal treatment

## Abstract

**Aim:**

To characterize the soft-tissue wall of remaining periodontal pockets for wound healing-related parameters versus healthy gingival crevices in the same individuals.

**Materials and methods:**

Gingival tissues collected from the diseased interface of pockets (GT biopsies) and from healthy gingival crevices (G biopsies) were subjected to RT^2^-profiler PCR Array for wound healing-related markers and network analysis of differentially expressed genes. Lymphangiogenesis-related gene expression was determined by qRT-PCR. The migration potential of mesenchymal stem cells isolated from GT biopsies (GT-MSCs) and G biopsies (G-MSCs) was evaluated by the scratch- and the transwell migration assays. The total collagen protein content was determined in GT-MSCs and G-MSCs homogenates.

**Results:**

Gene-ontology analysis on significantly upregulated genes expressed in GT biopsies revealed enrichment of several genes involved in processes related to matrix remodeling, collagen deposition, and integrin signaling. No significantly expressed genes were seen in G biopsies. Regarding lymphangiogenesis-related genes, GT biopsies demonstrated greater expression for *PROX1* than G biopsies (*p* = 0.05). Lower migration potential (*p* < 0.001), yet greater production of collagen protein (*p* = 0.05), was found for GT-MSCs over G-MSCs.

**Conclusion:**

Differential expression patterns of various molecular pathways in biopsies and cell cultures of diseased versus healthy gingival tissues indicate a potential of the former for tissue remodeling and repair.

**Clinical relevance:**

In the course of periodontitis, granulation tissue is formed within a periodontal defect in an attempt to reconstruct the site. Following treatment procedures periodontal granulation tissue remains inflamed but appears to retain healing potential.

**Supplementary information:**

The online version contains supplementary material available at 10.1007/s00784-023-05122-y.

## Introduction

Periodontitis is initiated by hyper-inflammatory responses within the tissues which in turn generate dysbiotic ecological changes within the microbial communities [1, 2]. Four distinct but overlapping phases (i.e., hemostasis, inflammation, proliferation, and maturation) characterize periodontal wound healing. In the presence of residual inflammation, granulation tissue (GT) is formed in an attempt to reconstruct the site, which is characterized by a highly dense network of blood arteries and capillaries, increased cellular density of fibroblasts and macrophages, and irregularly organized collagen fibers [3]. During regenerative periodontal surgery, GT is routinely excised [4, 5] aiming to promote wound healing and new attachment [6, 7], as it does no longer occupy the space that would be otherwise populated by regenerative cells. From a clinical standpoint, GT excision helps to control local inflammation and bleeding and thus, facilitates the debridement of a site, while it creates space for biomaterial grafting [8]. It should be noted that lower densities of immunocompetent cells were found in regenerated gingival units following resection of the pocket soft-tissue wall, compared to sites that this lining was left intact in situ [9].

However, there is controversy in the literature regarding the clinical benefits of resecting the pocket soft-tissue lining. Gingival curettage and GT removal in adjunction to scaling and root planing (SRP) failed to demonstrate additional clinical improvements over SRP alone [10]. Gingival curettage as a stand-alone procedure or when combined with SRP is not considered as a method of treatment [11] having little—if any—effect on gain in clinical attachment levels (CAL) [12]. Interestingly, it has been recently demonstrated that cells isolated from periodontal GT express embryonic stem cell markers [13] and appear to have osteogenic potential [14]. Remaining periodontal pockets following subgingival instrumentation, albeit inflamed, possess MSC-like cells with similar immunophenotypic characteristics to those isolated from healthy gingival crevices [15]. A pro-inflammatory milieu is found to suppress pluripotency, viability, and migration of human periodontal ligament stem cells (hPDLSCs), while these cell properties are promoted when inflammatory processes are regulated restoring the regenerative capacity of the cells [16].

Hence, a recent clinical trial studied the treatment outcomes of the Minimal Access Flap by retaining the soft-tissue wall of intrabony defects and thus, the multipotent stem cells that reside within it aiming to potentially promote tissue healing while minimizing gingival recession [17]. The current study aimed to characterize the soft-tissue wall lining residual periodontal pockets following step 1 and step 2 periodontal therapy, regarding wound healing-related parameters versus healthy gingival crevices in the same individuals.

## Materials and methods

### Patient selection

Convenience sampling of six pairs of tissue biopsies was used in the current study. Tissue biopsies were obtained in pairs (diseased and healthy) from six systemically healthy non-smokers with chronic periodontitis stage III, grade B (Appendix for the demographic characteristics of donors), who were scheduled to receive periodontal surgery at the Department of Preventive Dentistry, Periodontology and Implant Biology, Aristotle University of Thessaloniki (AUTh), Greece, in 2019. The study was approved by the School’s Ethical Committee (42/19-03-2019), and all participants signed an informed consent.

Inclusion criteria consisted of residual periodontal pockets of probing pocket depth (PPD) and clinical attachment levels > 5 mm with bleeding on probing and radiographic evidence of bone loss 3 months following steps 1 and 2 periodontal therapy. One tooth nearby the surgical field was free of clinical signs of periodontal inflammation (PPD ≤ 3 mm, absence of BOP, no radiographic evidence of bone loss). Exclusion criteria were > 65 years of age, history of systemic disease, compromised medical conditions requiring prophylactic antibiotic coverage, antibiotic therapy within the last 3 months, bisphosphonate medication, bone metabolic diseases, disorders that compromise wound healing, use of anti-inflammatory drugs, radiation, immunosuppressive therapy, narrow zone/absence of attached gingiva, pregnancy/lactation, smoking, and previous periodontal surgery.

### Collection of gingival tissue samples

Gingival tissue biopsies (six pairs) were harvested from remaining periodontal pockets during routine access-flap operation and from the gingival crevice of a healthy tooth that was non-adjacent to the periodontal defect but in proximity to the surgical field. Full-thickness gingival flaps were reflected using strictly intrasulcular incisions coupled with an internal bevel incision of 2 mm thickness for tissue uptake. The collar of the soft tissues (5 × 2 mm), which was demarcated by the two parallel incisions connected with a perpendicular incision, included the sulcular and attached epithelium with a thin layer of connective tissue also including the GT that lined the osseous defect. The tissue biopsy was submerged in RNA later and was stored at − 80 °C until further processed. The three pairs of biopsies were used for gene expression analysis (RT^2^-profiler PCR Array, lymphangiogenesis gene expression), while the other three for cell cultures.

### RNA isolation and quality control

The RNA isolated in the three pairs of biopsies (diseased and healthy from three individuals) passed through quality controls as described in detail in the Appendix.

###  Real-time quantitative reverse-transcription polymerase chain reaction (qRT-PCR)

Tissue specimens were analyzed by qRT-PCR for gene expression of lymphatic genes; vascular endothelial growth factor C (*VEGF-C*), homeobox transcription factor Prox1 (*PROX-1*), the lymphatic vessel endothelial hyaluronan receptor-1 (*LYVE-1*), podoplanin (*PDPN*), and C-C Motif Chemokine Ligand 21 (*CCL21*). In more detail, RNA extracted from three pairs of biopsies (diseased (D) and healthy (C)) was used for reverse transcription using the QuantiTect Reverse Transcription Kit (Qiagen, Hilden, Germany). The full protocol and nucleotide sequence of the associated genes are reported in the Appendix.

### RT^2^ profiler PCR Array

The wound healing RT^2^ profiler PCR Array (PAHS-121ZA, Qiagen) was used to evaluate the wound healing-related genes. The PCR reactions were performed using FastStart SYBR green master mix (Rox, Sigma-Aldrich, USA) and CFX-96, C100 Thermal Cycler. ACTB was used as housekeeping gene. Fold changes of gene expression were calculated using the ΔΔ_CT_ method [18] and inherent scaling functions in R (version 3.6.2.).

### Mesenchymal stem cell culture

In addition, MSCs isolated from GT biopsies (N_GT-MSCs_ = 3) and the analogous specimens from disease-free gingival crevices (N_G-MSCs_ = 3) were expanded at 37 °C in 5% CO_2_ in alpha-minimum essential medium (Life Technologies, Thermo Fisher Scientific, Paisley, UK) supplemented with 15% fetal bovine serum (Life Technologies), 100 units/mL of penicillin, 100 mg/mL of streptomycin, and 0.25 mg/ mL of amphotericin-B (all from Life Technologies). Cell cultures were established using the enzymatic dissociation method, as previously described [19].

### Scratch migration assay

The migration potential of the GT-MSCs and G-MSCs was assessed by the scratch assay [20]. Briefly, GT-MSCs and G-MSCs were seeded in 6-well plates (5 × 10^5^ cells/well) in CCM, and after reaching 90% confluency, CCM was changed to a-MEM with 1% FBS to induce serum deprivation and an even scratch was performed on the monolayer of cells by using a p200 and a p1000 pipette tip (Axygen Corning, NY, USA). The scratch was observed under an inverted microscope and photographs were taken at baseline (*t*_0_ after scratch), 12 h, 24 h, 30 h, 36 h, and 48 h in order to determine whether GT-MSCs or G-MSCs migrated faster to “close” the generated gap. The scratch width was measured by an inverted microscope equipped with a digital camera (Zeiss Axiovert 40, Carl Zeiss micro imaging, GmbH, Göttingen, Germany) and was used to determine the closest distance (*D*) between the two opposite sides of cells in five random fields of view. The migration (%) of cells (GT-MSCs and G-MSCs) was evaluated in relation to the initial width of the scratch based on the formula: migration [%] = Δ*D* (*t*_0_ − *t*_1)_/*t*_0_)*100. All samples were run in triplicate and so were the experiments.

### Transwell migration assay

The migration potential of GT-MSCs and G-MSCs at 24 and 30 h was quantified by the Transwell™ insert assay, based on the ability of the cells to move through a porous membrane. The protocol of this assay is reported in the Appendix. The number of migrated cells was counted twice at each field of view (FOV), and an average score was used. The percentage (%) of migration/invasion was determined based on the total number of cells perinsert. All samples were run in triplicate and so were the experiments.

### Total collagen assay

The Total Collagen Assay Kit (Perchlorate-Free) (Abcam 222942, Sigma-Aldrich, USA) (ab222942) was used to determine the collagen content in homogenates of GT-MSCs and G-MSCs cultures (Appendix for the protocol).

### Statistical analysis

#### RT^2^ profiler PCR array, gene profiling, and gene ontology analysis

The limma + voom pipeline was used to transform the data to the log scale and determine the dependence of the variance of log counts over the mean [21]. The *p*-values were adjusted based on the default FDR pipeline standards. Outcomes from the linear regression analysis and the calculation of adjusted *p*-values are demonstrated in the Appendix. Volcano plot was made with the *p*-value cutoff = 10e-2, and FC cutoff = 1.2. Gene ontology (GO) term analysis was completed by the EnrichR package after the *p*-value ranking of associated terms in Panther 2016 and GO Molecular Function 2018 databases [22].

#### Scratch migration assay, transwell migration assay, and total collagen assay

Statistical analyses for all cell culture assays were performed using the two-way analysis of variance (ANOVA). Comparisons between diseased and healthy samples at various timepoints were performed with Tukey’s post hoc test. The Prism 8.0 Software was used for the analyses (GraphPad, CA, USA), and the level of statistical significance was set at *p* < 0.05.

## Results

### Lymphangiogenesis-related genes

**Fig. 1:**
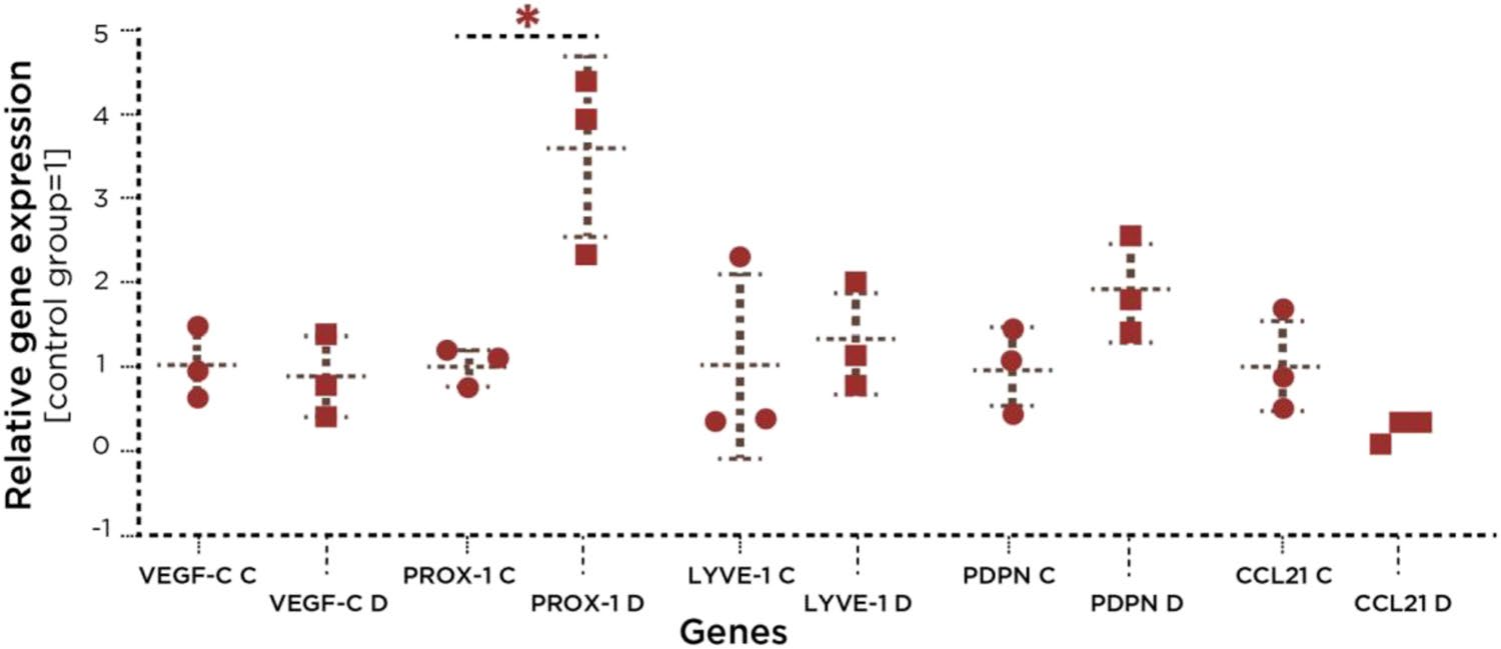
Lymphangiogenesis-related gene analysis. Real-time quantitative reverse transcription PCR analysis of the expression of lymphangiogenesis-related genes: vascular endothelial growth factor (*VEGF-C*), homeobox transcription factor Prox1 (*PROX-1*), lymphatic vessel endothelial hyaluronan receptor (*LYVE1*) podoplanin (*PDPN*), lymphatic vessel endothelial hyaluronan receptor (*LYVE1*), and C-C Motif Chemokine Ligand 21 (*CCL21*) in three donors. Asterisks indicate statistically significant differences between controls (C = healthy tissues) and diseased samples (D = diseased tissues) (*p < 0.05). Circle: control healthy samples, Square: diseased tissue samples

No significant differences were found between the diseased and healthy tissues in the expression of the following lymphagiogenesis-related genes: *VEGF-C*, *PDPN*, *LYVE1*, and *CCL21* (Fig. 1). However, in all three diseased tissue biopsies, significantly higher expression of the *PROX1* gene was noted over the healthy specimens (*p* < 0.05).

### Gene profiling analysis

**Fig. 2:**
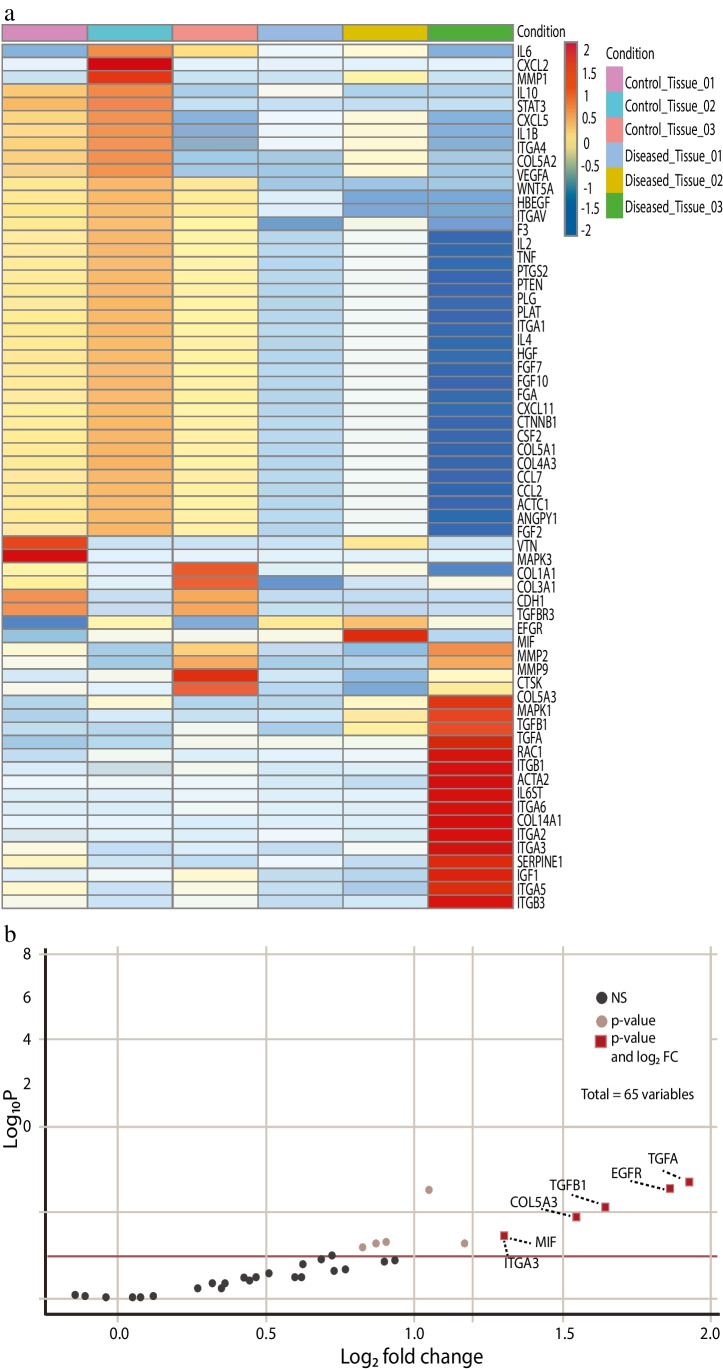
Gene profiling analysis related to wound healing. **a** Heatmap depicts the expression levels of quantitative PCR data between healthy (control) and diseased tissues of three patients in scale. Upregulated genes are indicated by red and downregulated genes are indicated by blue. **b** Volcano plot of most upregulated genes in healthy (control) versus diseased tissues. Differentially upregulated genes in the diseased tissues were picked by *p*-value cutoff = *p*-value cutoff = 10e-2, and FC cutoff = 1.2. The differentially upregulated genes comprised integrin subunit alpha-3 (*ITGA3*), macrophage migration inhibitory factor (*MIF*), collagen type-V alpha-3 chain (*COL5A3*), transforming growth factor-beta-1 (*TGF-β1*), epidermal growth factor receptor (*EGFR*), and transforming growth factor-alpha (*TGF-a*)

The heatmap revealed significant differences in the expression of wound healing-related genes between the diseased and healthy tissue biopsies (Fig. 2a). Of the 84 genes that were screened, 65 genes were expressed in both types of tissues. Among the upregulated genes, the expression levels of transforming growth factor-alpha (*TGF-a*), epidermal growth factor receptor (*EGFR*), transforming growth factor-beta-1 (*TGF-β1*), collagen type-V alpha-3 chain (*COL5A3*), macrophage migration inhibitory factor (*MIF*), and integrin subunit alpha-3 (*ITGA3*) were significantly greater in diseased tissues over the healthy specimens (*p*-value cutoff = 10e-2; FC cutoff = 1.2). The gene ontology (GO) term analysis was performed on the significantly upregulated genes expressed in the diseased versus the healthy tissues (*p*-value cutoff = 10e-2; FC cutoff = 1.2) and demonstrated enrichment of many biological processes in the former related to matrix remodeling, collagen deposition, and integrin signaling (Fig. 2b).

### Scratch migration assay

**Fig. 3:**
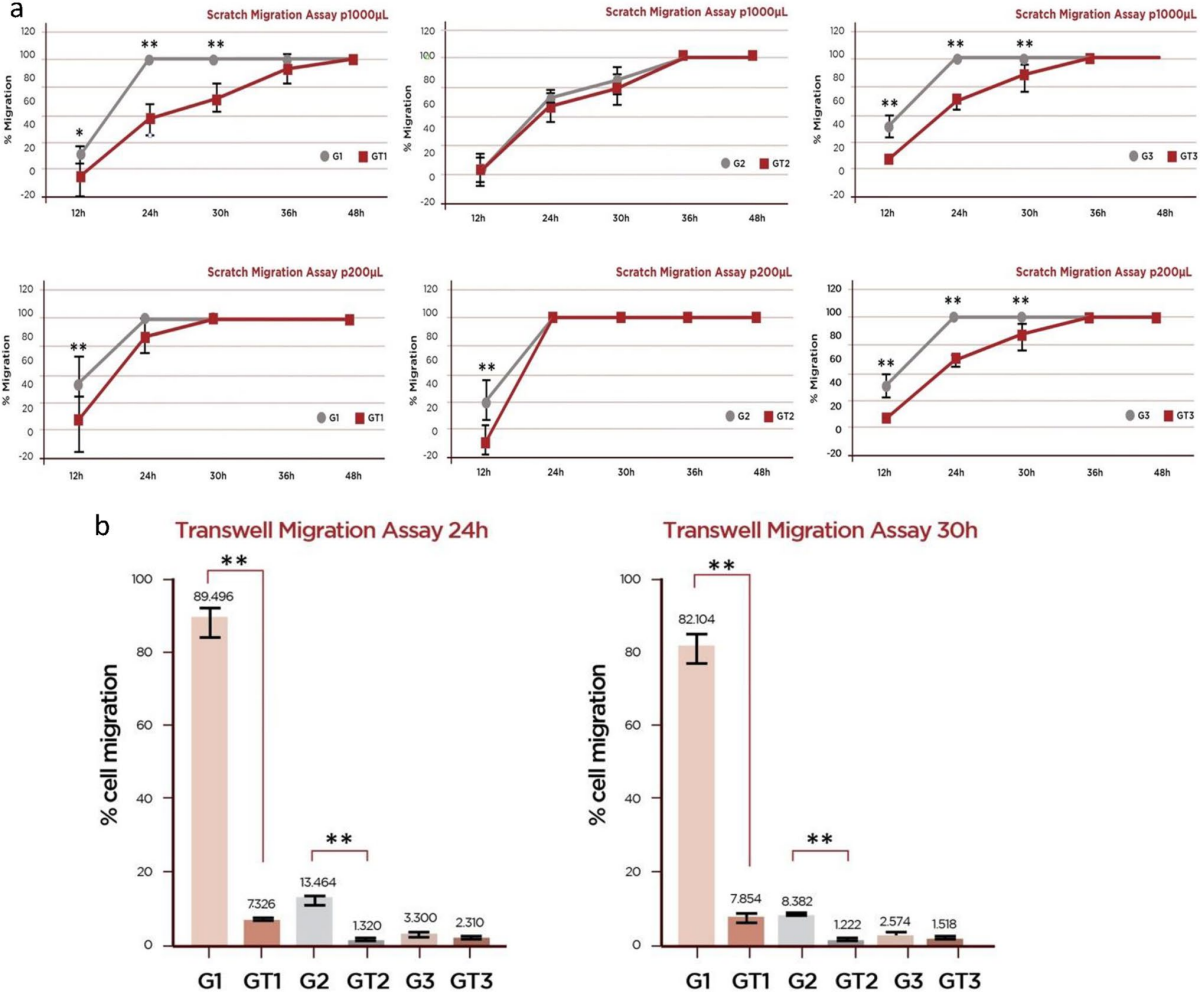
Migration assay. **a** Scratch migration assay: evaluation of the migration capacity of mesenchymal stem cells isolated in healthy gingival biopsies [G-MSCs (G)] and diseased gingival biopsies [GT-MSCs (GT)] of three donors by the scratch migration assay using a p1000 and a p200 pipette tip: vertical axis represents the percentage (%) of cell migration in relation to the distance the cells covered until the scratch finally closed (cell migration rate = 100%). Asterisks indicate statistically significant differences between cell cultures (*p < 0.05, **p < 0.01). **b** Transwell migration assay at 24 and 30 h: evaluation of the migration capacity of mesenchymal stem cells isolated in healthy gingival biopsies [G-MSCs (G)] and diseased gingival biopsies [GT-MSCs (GT)] of three donors at 24 h and 30 h by the Transwell.™ insert migration assay: vertical axis represents the percentage (%) of migrated cells. Asterisks indicate statistically significant differences between cultures (*p < 0.05, **p < 0.01)

A significantly lower migration rate was noted for GT-MSCs over G-MSCs at early timepoints significantly for donor 1 and donor 3 (*p* ≤ 0.05) (Fig. 3a), At p.1000, (mean %, sd) GT1 vs G1 at 12 h: 15.63 (14.08) vs 31.03 (8.09); at 24 h: 57.05 (10.76) vs 100; at 30 h: 71.76 (10.29) vs 100. The same pattern was also followed by donor 3 at 12 h: 27.95 (2.14) vs 51.77 (7.85); at 24 h: 69.36 (3.72) vs 100; at 30 h: 87.62 (8.76) vs 100 for GT3 vs G3, respectively. Similar findings were also found for p.200.

### Transwell migration assay

The 24-h observation timepoint revealed lower migration (%) of GT-MSCs than G-MSCs significantly for donors 1 and 2 (*p* < 0.01), in line with the 30-h timepoint (Fig. 3b).

### Total collagen assay

**Fig. 4:**
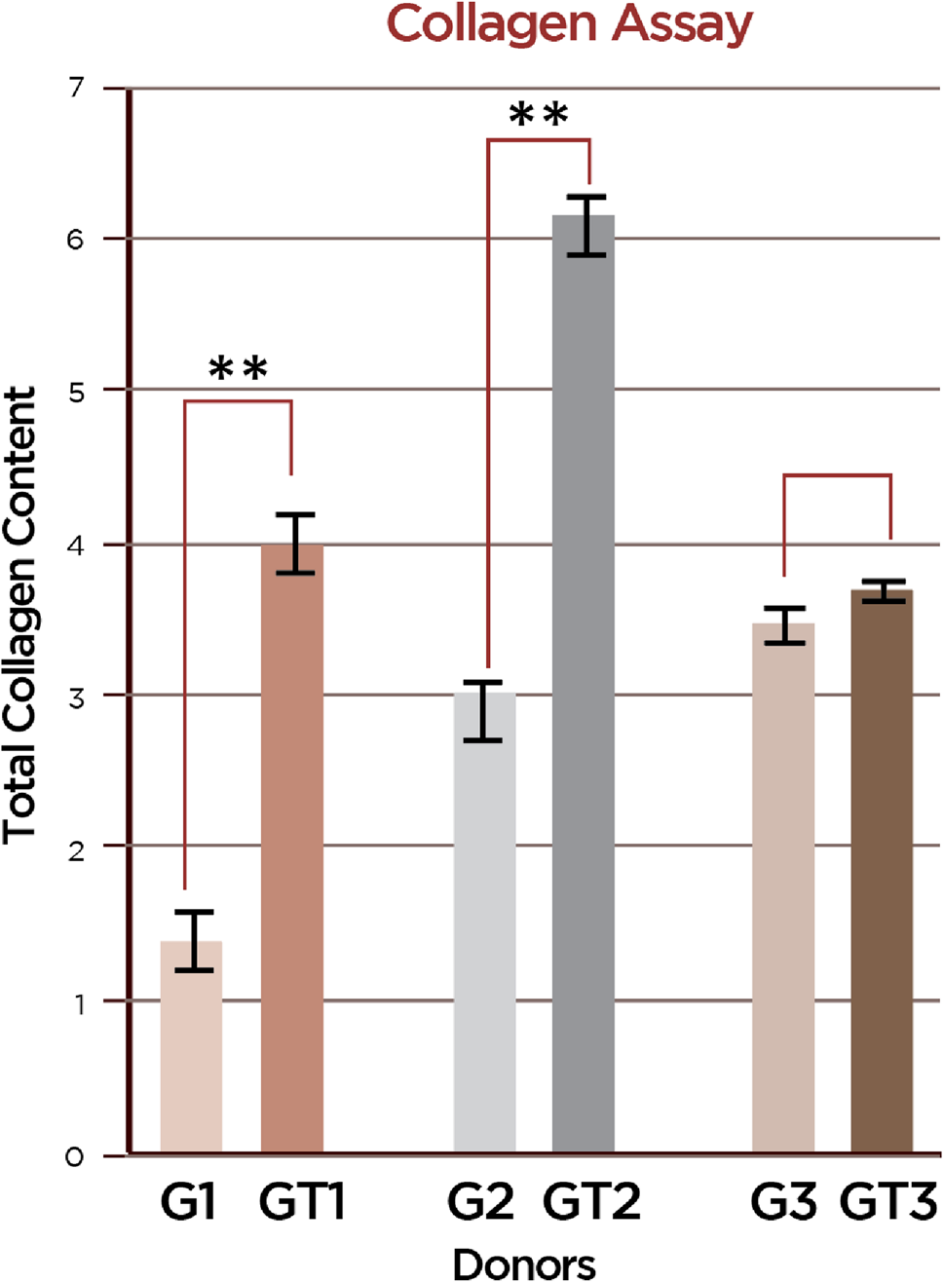
Total collagen assay. Evaluation of the collagen synthesis capacity of mesenchymal stem cells isolated in healthy gingival biopsies [G-MSCs (G)] and diseased gingival biopsies [GT-MSCs (GT)] of three donors (total cell homogenates) by the Total Collagen Assay Kit: vertical axis represents the total collagen content of cells. Asterisks indicate statistically significant differences between cultures (*p < 0.05, **p < 0.01)

Total collagen production was greater by GT-MSCs than by G-MSCs, significantly for donors 1 and 2 (*p* < 0.05 for both; mean (sd) GT1 vs G1: 3.56 (0.85) vs 1.52 (0.85); GT2 vs G2: 6.13 (0.19) vs 3.01 (0.01)) (Fig. 4).

## Discussion

During inflammation, lymphatic vessels sprout and proliferate within the infiltrated tissues [23], to facilitate the homing of leukocytes, eliminate the antigens, and drain the interstitial fluid from the inflammatory site [24]. However, there is a void in the literature regarding the contribution of lymphangiogenesis in periodontal wound healing and bone loss [25]. The lymphangiogenesis-related genes *PROX1* [26], *LYVE1* [27], and *PDPN* [28] are mainly used to distinguish lymphatic vessels from blood vessels. Current findings demonstrated that these genes were expressed in diseased and healthy gingival biopsies indicating a lymphangiogenesis potential in both types of tissue. However, the significantly greater expression of *PROX1* found in diseased tissues implies the presence of residual inflammation that was also clinically evident. The lymphatic response is known to be long-lasting, even after the inflammatory challenge has been removed [23], and if inflammation reoccurs, the remaining expanded vessel network contributes to the initiation of adaptive immune responses [29]. Although an increased vessel network is associated with inflammation resolution, lymphangiogenesis has also been demonstrated to play a role in perpetuating chronic inflammatory disorders [30]. Thus, the responses of lymphatic endothelial cells within the chronically inflamed gingivae may shed light into periodontal pathogenesis and this remains to be further studied.

The gene profiling in the inflamed and non-inflamed gingival tissues demonstrated that out of the 84 study key genes that are central to wound healing, 65 were expressed in both inflamed and healthy gingival tissues. However, gene expression between the pair-matched tissue specimens significantly differed for six genes; *TGF-α*, *EGFR*, *TGF-β1*, *COL5A3*, *MIF*, and *ITGA3*. The *TGF-α* and *TGF-β1* are important growth factors in periodontal healing but they have distinct biological roles. Transforming growth factor-alpha acts as a ligand for the epidermal growth factor receptor (*EGFR*), triggering cell proliferation, differentiation, and development [31]. Transforming growth factor-beta-1 promotes the production of GT by enhancing the expression of extracellular matrix (ECM)-related genes such as fibronectin, fibronectin receptor, collagen, and protease inhibitors [32]. It is also implicated in the recruitment of inflammatory cells and enhancement of tissue debridement by macrophages [32, 33]. In line with current findings, *TGF-β1* mRNA expression has been found higher in gingival tissue obtained from patients with periodontitis when compared to tissue harvested from subjects free of periodontitis [34]. In the current study, macrophage migration inhibitory factor (*MIF*) was significantly upregulated in GT samples. Macrophage inhibitory factor is a pro-inflammatory mediator [35] and has been shown to contribute to both the inflammatory and migration/proliferation phases of wound healing [36]. In the inflammatory phase, chronic expression of *MIF* would serve to attenuate wound healing progression due to its potent inflammatory potential. Therapeutic targeting of *MIF* might be promising in diseases with impaired/unbalanced wound healing [36]. However, due to its pro-proliferative activity, *MIF* expression may contribute to improving wound healing by exerting its effects on fibroblasts [36], and this is central to periodontal regeneration. The current study indicated that *COL5A3* gene was significantly upregulated in GT samples. Collagen-V, a protein produced by the *COL5A* gene, is the connector between the basement membrane and stroma and promotes cell attachment and migration [37]. It has been shown to regulate the initiation of collagen fibril assembly, making it an essential regulator of fibril formation and matrix organization [38]. A recent study has demonstrated that matrix containing *COL5A3* may be required for the initiation of wound healing [39]. In addition, *ITGA3* was significantly upregulated in GT samples. Integrins are heterodimeric transmembrane proteins that play significant roles in cell proliferation, differentiation, cell-cell attachment, adhesion, and signal transduction between the cell and the ECM during developmental and pathological processes [40]. They can also bind to other ECM proteins such as vitronectin, fibronectin, laminin, and collagen because of their adhesive nature [41]. In conclusion, the upregulated genes in the diseased tissues were involved in the proliferation phases of wound healing by collagen deposition, matrix remodeling, and integrin signaling indicating that despite the remaining inflammatory stimulus, the infiltrated tissues are in a process of restoration, possibly for prolonged time.

This study also investigated the properties of isolated MSCs in diseased versus healthy tissue biopsies. The scratch migration assay comprises a semi-quantitative measurement of migration of monolayer cultured MSCs, indicating their capacity to proliferate and bridge the generated gap in vitro. It should be noted that due to the manual technique employed to generate the scratch, there might be variation in the depth, size, and borders of the scratch. This limitation was overcome by the transwell migration assay which sought to reveal the actual potential of the cells to migrate towards a chemoattractant agent. Current data demonstrated a lower proliferation rate of MSCs isolated from the diseased gingival tissues over the healthy tissues and a tendency for lower migration rate of the former than the latter cells. This finding is in partial agreement with previous data [42], which demonstrated that the population doubling ability of healthy gingival MSCs was greater than that of inflamed gingival MSCs, indicating that the inflammatory state of gingival tissues has an impact on the clonal expansion of residing stem/progenitor cells.

Of interest is the interpretation of current findings from the angle of collagen apposition. In line with the gene profiling analysis which demonstrated the upregulation of genes related to collagen synthesis, greater production of collagen was found by the MSCs isolated in the diseased biopsy over the healthy gingival tissue. It might well be that MSCs in GT are committed to collagen production and matrix synthesis rather than migration and/or proliferation. During wound healing, the fibroblasts proliferate to produce ECM and restore the damaged tissues. This role is seemingly “turned on” in inflamed tissues as an attempt to restore the tissues; however, with GT formation, the site remains unresolved in the long-term unless treatment reforms the site and successfully controls inflammation. However, consistent with the current study, other studies in animal models [43–45] and in humans [46] have shown that GT has higher levels of collagen than non-inflamed tissues in the early stages of wound healing. These data do not reflect on the organization of the collagen bundles within the tissues and possibly indicate an effort of the host to repair the damaged tissue by increased production of collagen.

Interestingly, a clinical study demonstrated that non-resective surgical approaches that retained the soft-tissue lining of the pocket led to greater residual lesions with greater densities of immunocompetent cells in the regenerated gingival tissues at 6 months [9], implying an inferior healing outcome. However, a relatively short time frame for re-evaluation should be considered in that study design [47]. The current study sheds light into the wound healing process of gingival tissues with remaining inflammation and follows up previous studies on this topic which investigated the outcomes of preserving the pocket gingival lining [15, 17]. This in vitro study was conducted on tissue biopsies retrieved from six non-smoking donors, and despite a relatively small sample size of biopsies (three pairs of biopsies for each arm of the experiment), there was homogeneity in the donors in terms of a similar periodontal clinical status. Because of the small quantities of specimens, three donors were used for the gene profiling analysis and the remaining three for the cell culture assays. However, despite the different specimens used in the two experimental arms of the study, there was a consistent trend across all donors. It should be noted that the samples of health versus disease were pair-matched, to exclude any biological variability across donors. Further research is suggested to determine the role that the upregulated genes related to wound healing might play in a successful treatment outcome and whether the different components of the ECM might play a role in inflammation control and maturation of the GT into connective tissue.

## Conclusion

Current findings indicate that the soft-tissue wall of remaining periodontal pockets albeit inflamed plays a role in wound healing and periodontal reconstruction. This is evidenced by the finding that several biological processes (i.e., collagen deposition, matrix remodeling, and integrin signaling) were enhanced within this zone of tissues that is in close proximity to the bacterial interface and thus, to the inflammatory stimulus. Unsurprisingly, upregulation of *PROX-1* gene indicates that lymphangiogenesis is more evident within tissues with remaining inflammation. The MSCs isolated in the inflamed tissues of remaining periodontal pockets showed a potential for lower migration and proliferation but for greater synthesis of collagen possibly as an attempt to reconstruct the damaged site. Thus, the effective control of inflammation by periodontal treatment at early stages is of great importance to reforming the site and potentially leads to the maturation of what appears to be disturbed healing tissue.

## Supplementary information

Below is the link to the electronic supplementary material.

Supplementary file1 (DOCX 36 KB)

## Data Availability

The data that support the findings of this study are available in the supplementary material of this article and can also be available from the corresponding author upon reasonable request.
