# Supplementary material for: Characterization of the soft-tissue wall lining residual periodontal pockets and implications in periodontal wound healing
Source: Clin Oral Investig. 2023 Jul 24;27(9):5031–40. doi: 10.1007/s00784-023-05122-y (PMC10492763; doi:10.1007/s00784-023-05122-y)
Supplement: Supplementary file 1 — Supplementary file1 (DOCX 36 KB) [file 784_2023_5122_MOESM1_ESM.docx]

**Appendix**

**Characterization of the soft-tissue wall lining residual periodontal pockets and implications in periodontal wound healing**

Evangelia Gousopoulou^1^, Athina Bakopoulou^2^, Dimitrios Laskaris^3^, Epameinondas Gousopoulos^4^, Danae A. Apatzidou^1,*^

^1^ Department of Preventive Dentistry, Periodontology & Implant Biology, School of Dentistry, Faculty of Health Sciences, Aristotle University of Thessaloniki (A.U.T.H.), GR-54124, Thessaloniki, Greece

^2^ Department of Prosthodontics, School of Dentistry, Faculty of Health Sciences, Aristotle University of Thessaloniki (A.U.T.H.), GR-54124, Thessaloniki, Greece

^3^ Department of Molecular Pathology, Netherlands Cancer Institute, 1066CX, Amsterdam, Netherlands

^4^ Department of Plastic Surgery and Hand Surgery, University Hospital Zurich, 8091 Zurich, Switzerland

*Corresponding author:

Danae Apatzidou

Department of Preventive Dentistry, Periodontology & Implant Biology, School of Dentistry, Faculty of Health Sciences, Aristotle University of Thessaloniki (A.U.T.H.), GR-54124, Thessaloniki, Greece

E-mail address: [perioapatzidou@yahoo.gr](mailto:perioapatzidou@yahoo.gr), [dapatzidou@dent.auth.gr](mailto:dapatzidou@dent.auth.gr)

| Donors | Gender | Age | Experimental methods | Samples |
| --- | --- | --- | --- | --- |
| #1 | male | 61 | Gene expression of lymphatic genes, RT^2^ profiler PCR Array | Healthy gingival tissue (C)  Diseased gingival tissue (D) |
| #2 | male | 51 |  |  |
| #3 | male | 64 |  |  |
| #4 | male | 41 | Scratch migration assay, Transwell migration assay, Total collagen assay | MSCs isolated in healthy gingival tissue  MSCs isolated in diseased gingival tissue |
| #5 | female | 48 |  |  |
| #6 | female | 50 |  |  |

**Demographic characteristics of donors**

**RNA isolation and quality control**

RNA was extracted from gingival tissue biopsies, using TRIzol (Ambion) after mechanical homogenization in a TissueLyser (QIAGEN), followed by purification with the NucleoSpin RNA Kit according to the manufacturer’s protocol (Macherey-Nagel). Evaluation of RNA integrity was performed using the Agilent RNA 6000 Nano kit (Agilent Technologies).

**Quantitative reverse-transcription polymerase chain reaction (qRT-PCR)**

Tissue specimens were analyzed by qRT-PCR for gene expression of lymphatic genes. In more detail, 1g of RNA extracted from the three pairs of biopsies (3 control healthy; 3 diseased) was used for reverse transcription using the QuantiTect Reverse Transcription Kit (Qiagen, Hilden, Germany). The RNA samples used had a A260/A280 ratio of ≥1.5. Quantitative RT-PCR was performed on a CFX 96 C100 Thermal Cycler. The FastStart SYBR green master mix (Rox, Sigma-Aldrich, USA) was used for target amplification and quantification of the following lymphatic cell marker gene expression: vascular endothelial growth factor C (VEGF-C), homeobox transcription factor Prox1 (PROX-1), the lymphatic vessel endothelial hyaluronan receptor-1 (LYVE-1), podoplanin (PDPN) and C-C Motif Chemokine Ligand 21 (CCL21). Amplification conditions were as follows: initial incubation step of 5min at 95^◦^C to activate HotStarTaq DNA polymerase and 40 cycles of denaturation (at 95^◦^C for 5s) and combined annealing/extension (at 60^◦^C for 10s). Afterwards, a melting curve was recorded and exported raw data were analyzed. Baseline correction was carried out and mean amplification efficiencies per amplicon as well as CT values for each sample were calculated using LinRegPCR [1]. Expression of six candidate reference genes was determined, including beta actin (ACTB), beta-2-microglobulin (B2M), glyceraldehyde-3-phosphate dehydrogenase (GAPDH), 18S ribosomal RNA (RRN18S), succinate dehydrogenase flavoprotein subunit (SDHA2) and tyrosine 3-monooxygenase/tryptophan 5-monooxygenase activation protein zeta (YWHAZ). Of the six genes, the two most stable genes were identified by geNorm [2] and served as the positive controls of the assay. Subsequently, fold changes in gene expression were calculated according to the efficiency adjusted delta delta-CT method [3]. The paired-samples t-test compared lymphangiogenesis related gene expression in healthy tissue originating from a healthy periodontal crevice and diseased tissues lining the soft-tissue wall of remaining periodontal pockets. Analysis was performed with Prism 8.0 Software (GraphPad, CA, USA) and the level of statistical significance was set at * p < 0.05.

**Transwell Migration Assay**

Τhe quantitative assessment of the migratory potential of gingival mesenchymal stem cells (G-MSCs) and granulation tissue MSCs (GT-MSCs) was performed at two time-points (24- and 30-h) by the Transwell^TM^ insert assay, which is based on the ability of cells to move through a porous membrane. Transwell inserts were previously coated with 50 μg/cm^2^ collagen type-I solution (Collagen I Rat tail, Corning, NY, USA) for 1 h at 37°C, 5% CO_2_, and 100% humidity, double washed with PBS (Gibco™ Grand Island, NY, USA) and were dried before cell seeding. G-MSCs and GT-MSCs were first cultured in flasks (75 cm^2^) with CCM and when cell cultures reached 80–85% confluence, CCM was changed to a-MEM with 1% FBS for 18-20 h to induce serum deprivation. Afterwards, 10^5^ cells/100 μl a-MEM with 1% FBS were seeded into the polyester transwell (PET) inserts of 24-well plates with an 8 μm membrane pore size (Corning, Life Sciences Ltd). In the lower chamber of the 24-well plates 1 ml/well CCM (with 15% FBS as chemoattractant) was added.

For each time-point, transwell inserts were stained and fixed according to the manufacturer’s instructions. Briefly, culture medium was aspirated, and the inserts were washed twice with PBS. The upper surface of the membrane was gently swabbed to remove the cells that failed to migrate through the membrane. Inserts were then fixed with 4% paraformaldehyde for 45 min at room temperature (RT) and afterwards, the fixative was removed, and inserts were washed twice with dH_2_O (deionized water). Subsequently, dH_2_O was removed and the inserts were stained with crystal violet staining solution (0.5%w/v) for 10 min at RT. Afterwards, the stain was removed and washed twice with dH_2_O. Photographs of the stained cells were captured from at least five random fields of view (×20) for each membrane under an inverted microscope equipped with a digital camera (Zeiss Axiovert 40, Carl Zeiss micro imaging, GmbH, Göttingen, Germany). Finally, the number of migrated cells were counted twice in each field of view (FOV) and the average of migrated cells was calculated. Once the total number of cells *per* insert was determined, the percentage of migration/invasion was calculated. All samples were run in triplicates and the experiments were repeated three times.

**Total Collagen Assay**

Total collagen from G-MSCs and GT-MSCs cultures was measured using Total Collagen Assay Kit (Perchlorate-Free) (Abcam 222942, Sigma-Aldrich, USA). G-MSCs and GT-MSCs were first cultured in cell culture plates (100mm x 20mm) with CCM for 25 days. Then, each plate was washed with PBS and cells were collected using a cell scraper. The cells were centrifuged with a Beckman JS-5.2 rotor at 4°C. To homogenize the samples, the pellets were minced into small pieces using sterile surgical scalpel blades No. 11. The pellets were resuspended into 500 μL of distilled water and all samples were homogenized by vortex mixing. Then samples were transferred to -80°C for 20 minutes and afterwards to room temperature for 15 minutes. Protein levels in the collected supernatants were measured in duplicate using a Pierce BCA Protein Assay Kit (Cat. No. 23225, Thermo Fisher Scientific).

Samples were mixed with equal volume of 10 N concentrated NaOH in pressure-tight, screw-capped polypropylene vial and hydrolyzed at 120°C for 1 hour. Then vials were cooled on ice and the hydrolysates were neutralized by adding an equivalent volume of 10 N concentrated HCl to NaOH added. All vials were homogenized through vortex and centrifuged to pellet any insoluble debris that may remain following hydrolysis. Supernatants were collected and transferred to new tubes kept on ice. A standard curve dilution was generated using 1 mg/mL hydrolyzed Collagen I.

The sample hydrolysates and standard curve wells were evaporated to dryness by heating the plate at 65°C on a hot plate/dry heat block or microplate incubator. Following evaporation of the hydrolysates, a crystalline residue was left in the well. Then 100 μL of Oxidation Mix was prepared for each reaction.

The absorbance values were measured at 550nm and compared to those of the standard curve to determine the concentration of hydrolyzed collagen I in the samples. The concentrations of total collagen in homogenates were determined by the amount of hydrolyzed collagen multiplied by the optimal sample dilution fold based on the manufacturer instructions during the generation of the hydrolysate.

**Statistical analysis**

**RT^2^ profiler PCR Array, Gene Profiling and Gene Ontology Analysis**

| gene id | logFC | Average expression | t | P.value | adj.P.value | B |
| --- | --- | --- | --- | --- | --- | --- |
| ACTA2 | 0,93581813 | 15,56857 | 1,52334677 | 0,128887318 | 0,36424677 | -4,79456 |
| ACTC1 | 0,44726894 | 14,52688 | 0,95084118 | 0,342568039 | 0,40485314 | -5,682887 |
| ANGPT1 | 0,44726894 | 14,52688 | 0,95084118 | 0,342568039 | 0,40485314 | -5,682887 |
| CCL2 | 0,44726894 | 14,52688 | 0,95084118 | 0,342568039 | 0,40485314 | -5,682887 |
| CCL7 | 0,44726894 | 14,52688 | 0,95084118 | 0,342568039 | 0,40485314 | -5,682887 |
| CDM1 | 0,43163706 | 14,19481 | 0,9755635 | 0,330187607 | 0,40485314 | -5,716164 |
| COL14A1 | 1,04824008 | 13,85774 | 3,00106187 | 0,00295195 | 0,01918768 | -2,018669 |
| COL1A1 | 0,12261077 | 15,22107 | 0,20791884 | 0,835455111 | 0,87588036 | -5,880687 |
| COL1A2 | 0,61786318 | 15,28093 | 1,06357893 | 0,288506293 | 0,40485314 | -5,389426 |
| COL3A1 | 0,44726894 | 14,52688 | 0,95084118 | 0,342568039 | 0,40485314 | -5,682887 |
| COL4A3 | 0,44726894 | 14,52688 | 0,95084118 | 0,342568039 | 0,40485314 | -5,682887 |
| COL5A1 | 0,5077948 | 14,32783 | 1,14681254 | 0,252513369 | 0,40485314 | -5,541507 |
| COL5A2 | 1,54651111 | 15,86326 | 2,51514985 | 0,012501904 | 0,06771864 | -2,950078 |
| COL5A3 | 0,44726894 | 14,52688 | 0,95084118 | 0,342568039 | 0,40485314 | -5,682887 |
| CSF2 | 0,44726894 | 14,52688 | 0,95084118 | 0,342568039 | 0,40485314 | -5,682887 |
| CTNNB1 | 0,87304494 | 14,16061 | 1,9371809 | 0,05380637 | 0,21858838 | -4,360764 |
| CTSG | 0,34912727 | 15,37889 | 0,60288901 | 0,547108032 | 0,61313831 | -5,748735 |
| CT5K | 0,44726894 | 14,52688 | 0,95084118 | 0,342568039 | 0,40485314 | -5,682887 |
| CXCL11 | -0,11075802 | 14,47448 | -0,22222154 | 0,824315886 | 0,87588036 | -6,034939 |
| CXCL2 | 0,72252358 | 14,40367 | 1,64146852 | 0,10190922 | 0,3154333 | -4,886418 |
| CXCL5 | 1,86136909 | 15,8421 | 3,02838236 | 0,002706021 | 0,01918768 | -1,640485 |
| EGFR | 0,26991654 | 14,45596 | 0,56914675 | 0,56974789 | 0,62768835 | -5,951398 |
| F3 | 0,44726894 | 14,52688 | 0,95084118 | 0,342568039 | 0,40485314 | -5,682887 |
| FGA | 0,44726894 | 14,52688 | 0,95084118 | 0,342568039 | 0,40485314 | -5,682887 |
| FGF10 | 0,46952198 | 14,49258 | 1,01954153 | 0,308893684 | 0,40485314 | -5,637673 |
| FGF2 | 0,44726894 | 14,52688 | 0,95084118 | 0,342568039 | 0,40485314 | -5,682887 |
| FGF7 | -0,03967989 | 14,77585 | -0,07641253 | 0,93914967 | 0,93914967 | -6,016818 |
| HBEGF | 0,44726894 | 14,52688 | 0,95084118 | 0,342568039 | 0,40485314 | -5,682887 |
| HGF | 0,72927498 | 15,4989 | 1,20468716 | 0,229419522 | 0,40485314 | -5,206786 |
| IGF1 | 0,62663003 | 14,29278 | 1,42587668 | 0,155103451 | 0,40326897 | -5,204471 |
| IL10 | 0,72252358 | 14,40367 | 1,64146852 | 0,10190922 | 0,3154333 | -4,886418 |
| IL18 | 0,44726894 | 14,52688 | 0,95084118 | 0,342568039 | 0,40485314 | -5,682887 |
| IL2 | 0,44726894 | 14,52688 | 0,95084118 | 0,342568039 | 0,40485314 | -5,682887 |
| IL4 | 0,82890893 | 14,39948 | 1,87582477 | 0,061799783 | 0,22316588 | -4,488521 |
| IL6 | 1,04824008 | 13,85774 | 3,00106187 | 0,00295195 | 0,01918768 | -2,018669 |
| IL6ST | 0,44726894 | 14,52688 | 0,95084118 | 0,342568039 | 0,40485314 | -5,682887 |
| ITGA1 | 0,72252358 | 14,40367 | 1,64146852 | 0,10190922 | 0,3154333 | -5,682887 |
| ITGA2 | 1,04824008 | 13,85774 | 3,00106187 | 0,00295195 | 0,01918768 | -2,018669 |
| ITGA3 | 1,29846228 | 15,69356 | 2,11167122 | 0,035668865 | 0,16560545 | -3,80941 |
| ITGA4 | 0,90068894 | 15,37021 | 1,4964586 | 0,135747077 | 0,36764833 | -4,847522 |
| ITGA5 | 1,04824008 | 13,85774 | 3,00106187 | 0,00295195 | 0,01918768 | -2,018669 |
| ITGA6 | 0,36155729 | 14,42274 | 0,76856767 | 0,442847543 | 0,51401947 | -5,831991 |
| ITGAV | 1,1725818 | 15,69842 | 1,90713613 | 0,057604985 | 0,22025435 | -4,187855 |
| ITGB1 | 1,04824008 | 13,85774 | 3,00106187 | 0,00295195 | 0,01918768 | -2,018669 |
| ITGB3 | 1,04824008 | 13,85774 | 3,00106187 | 0,00295195 | 0,01918768 | -2,018669 |
| MAPK1 | 0,68938698 | 14,04001 | 1,57995812 | 0,115332223 | 0,34075429 | -4,988812 |
| MPAK3 | 1,30638603 | 15,53895 | 2,12615632 | 0,034432591 | 0,16560545 | -3,781598 |
| MIF | 0,90629363 | 14,17877 | 1,96305021 | 0,050706321 | 0,21858838 | -4,293546 |
| MMP1 | 0,76839584 | 15,50438 | 1,26781851 | 0,20599696 | 0,40485314 | -5,133806 |
| MMP2 | 0,76839584 | 15,50438 | 1,26781851 | 0,20599696 | 0,40485314 | -5,133806 |
| MMP9 | 0,60500788 | 14,93871 | 1,03650767 | 0,300928397 | 0,40485314 | -5,411746 |
| PLAT | 0,44726894 | 14,52688 | 0,95084118 | 0,342568039 | 0,40485314 | -5,682887 |
| PEG | 0,44726894 | 14,52688 | 0,95084118 | 0,342568039 | 0,40485314 | -5,682887 |
| PTEN | 0,44726894 | 14,52688 | 0,95084118 | 0,342568039 | 0,40485314 | -5,682887 |
| PTGS2 | 0,44726894 | 14,52688 | 0,95084118 | 0,342568039 | 0,40485314 | -5,682887 |
| RAC1 | 1,04824008 | 13,85774 | 3,00106187 | 0,00295195 | 0,01918768 | -2,018669 |
| SERPINE1 | 1,04824008 | 13,85774 | 3,00106187 | 0,00295195 | 0,01918768 | -2,018669 |
| STAT3 | 0,32035815 | 14,21694 | 0,75091256 | 0,453384356 | 0,51701725 | -5,935911 |
| TGFA | 1,92702565 | 15,80145 | 3,13685303 | 0,001904143 | 0,01918768 | -1,332729 |
| TGFB3 | 1,64608523 | 15,60671 | 2,68053648 | 0,007820187 | 0,0462102 | -2,555029 |
| TGFBR3 | 0,43163706 | 14,19481 | 0,9755635 | 0,330187607 | 0,40485314 | -5,716164 |
| TNF | 0,44726894 | 14,52688 | 0,95084118 | 0,342568039 | 0,40485314 | -5,716164 |
| VEGFA | 0,5077948 | 14,32783 | 1,14681254 | 0,252513369 | 0,40485314 | -5,541507 |
| VTN | 0,05126937 | 15,06384 | 0,09369087 | 0,925426879 | 0,93914967 | -5,966254 |
| WNT5A | 0,0760671 | 14,35182 | 0,16569678 | 0,868524351 | 0,89609655 | -6,123346 |

**References**

1. Ruijter, J. M., Ramakers, C., Hoogaars, W. M., Karlen, Y., Bakker, O., van den Hoff, M. J., & Moorman, A. F. (2009). Amplification efficiency: linking baseline and bias in the analysis of quantitative PCR data. *Nucleic acids research*, *37*(6), e45. <https://doi.org/10.1093/nar/gkp045>
2. Vandesompele, J., De Preter, K., Pattyn, F., Poppe, B., Van Roy, N., De Paepe, A., & Speleman, F. (2002). *Genome Biology, 3(7), research0034.1.* doi:10.1186/gb-2002-3-7-research0034
3. Pfaffl M. W. (2001). A new mathematical model for relative quantification in real-time RT-PCR. *Nucleic acids research*, *29*(9), e45. <https://doi.org/10.1093/nar/29.9.e45>
